# Prediction of Response to Preoperative Neoadjuvant Chemotherapy in Locally Advanced Cervical Cancer Using Multicenter CT-Based Radiomic Analysis

**DOI:** 10.3389/fonc.2020.00077

**Published:** 2020-02-04

**Authors:** Xin Tian, Caixia Sun, Zhenyu Liu, Weili Li, Hui Duan, Lu Wang, Huijian Fan, Mingwei Li, Pengfei Li, Lihui Wang, Ping Liu, Jie Tian, Chunlin Chen

**Affiliations:** ^1^Department of Gynaecology and Obstetrics, Nanfang Hospital, Southern Medical University, Guangzhou, China; ^2^CAS Key Laboratory of Molecular Imaging, Institute of Automation, Chinese Academy of Sciences, Beijing, China; ^3^Key Laboratory of Intelligent Medical Image Analysis and Precise Diagnosis of Guizhou Province, School of Computer Science and Technology, Guizhou University, Guiyang, China; ^4^School of Artificial Intelligence, University of Chinese Academy of Sciences, Beijing, China; ^5^Beijing Advanced Innovation Center for Big Data-Based Precision Medicine, Beihang University, Beijing, China

**Keywords:** locally advanced cervical cancer (LACC), radiomics, neoadjuvant chemotherapy, response prediction, CT

## Abstract

**Objective:** To investigate whether pre-treatment CT-derived radiomic features could be applied for prediction of clinical response to neoadjuvant chemotherapy (NACT) in locally advanced cervical cancer (LACC).

**Patients and Methods:** Two hundred and seventy-seven LACC patients treated with NACT followed by surgery/radiotherapy were included in this multi-institution retrospective study. One thousand and ninety-four radiomic features were extracted from venous contrast enhanced and non-enhanced CT imaging for each patient. Five combined methods of feature selection were used to reduce dimension of features. Radiomics signature was constructed by Random Forest (RF) method in a primary cohort of 221 patients. A combined model incorporating radiomics signature with clinical factors was developed using multivariable logistic regression. Prediction performance was then tested in a validation cohort of 56 patients.

**Results:** Radiomics signature containing pre- and post-contrast imaging features can adequately distinguish chemotherapeutic responders from non-responders in both primary and validation cohorts [AUCs: 0.773 (95% CI, 0.701–0.845) and 0.816 (95% CI, 0.690-0.942), respectively] and remain relatively stable across centers. The combined model has a better predictive performance with an AUC of 0.803 (95% CI, 0.734–0.872) in the primary set and an AUC of 0.821 (95% CI, 0.697–0.946) in the validation set, compared to radiomics signature alone. Both models showed good discrimination, calibration.

**Conclusion:** Newly developed radiomic model provided an easy-to-use predictor of chemotherapeutic response with improved predictive ability, which might facilitate optimal treatment strategies tailored for individual LACC patients.

## Introduction

Locally advanced cervical cancer (LACC) suffer from high risks of treatment failure (1). Neoadjuvant chemotherapy (NACT) followed by radical hysterectomy is increasingly applied as alternative to standard chemoradiotherapy for LACC in some countries as NACT could shrink tumor volume and render unresectable tumors operable (2, 3). Recent evidence further revealed comparable oncological outcome between NACT-surgery and chemoradiotherapy for stage IB2-IIB cervical cancer, indicating optimal treatment decision relied upon quality of life (4). NACT is beneficial in controlling micro-metastasis and potentially improves patients' quality of life by preserving sexual capacity and ovarian function (5, 6). Moreover, pathological optimal responders could even exhibit survival benefit without need for post-operative adjuvant treatments (7, 8). However, chemotherapeutic non-responders suffer from disease progression and experience worse prognosis due to delay in definite therapies (9, 10). Therefore, clinical use of NACT for LACC should be more precise and individualized. Only chemotherapeutic responders can be candidates for preoperative NACT, whereas non-responders should directly undergo chemoradiotherapy avoiding ineffective therapies. Therefore, an overwhelming need to discover pretherapy predictors of NACT response to stratify LACC patients to appropriate therapies.

Immunohistochemical markers identified by measuring alterations in protein expression from tumor tissue samples (pre- and post-chemotherapy) are relatively unreliable due to lack of validation and conflicting results in various studies (11, 12). Some proposed genomic, proteomic predictive signature are limited by small sample size and high cost of examination (13, 14). Several pioneer studies demonstrated quantitative parameters derived from pre- and post-treatment functional imaging of LACC patients were powerful markers to non-invasively predict early therapeutic response to NACT (15, 16). These quantitative parameters allow for characterizing tumor biological process during NACT. However, post-treatment nature limits its extensive utility in therapy decision.

Radiomics, an emerging approach for image interpretation, can non-invasively predict treatment response solely by radiographic examination (17). Radiomics can profile tumor heterogeneity of different responders by extracting extensively quantitative features that reflect underlying pathophysiology from medical images (18). In fact, radiomics has proved to perform well in prognosis prediction of cervical cancer treated with chemoradiotherapy using PET/CT and MRI images (19). Moreover, pretherapy computed tomography (CT) radiomics also managed to predict response to neoadjuvant chemoradiotherapy for locally advanced rectal cancer (20). Furthermore, Li et al. pilot study successfully applied pre-treatment CT radiomics in predicting pathological response after neoadjuvant chemotherapy in locally advanced gastric cancers (21). In light of this, radiomics is promising for pre-therapy prediction of response to NACT for LACC.

CT is widely used for pre-treatment staging of cervical cancer (22, 23). Investigation regarding the feasibility of pretreatment CT-based radiomics for prediction of NACT response in LACC has not yet reported. Meantime, limited reproducibility in multi-center context remain a crucial bottleneck in widespread use of radiomics in clinic. Thus, the aim of the study is to establish and validate an effective pretherapy CT-based radiomic model for predicting response to NACT while testify stability and reproducibility of radiomic features and models on a multi-center basis.

## Materials and Methods

### Patients

**Figure 1:**
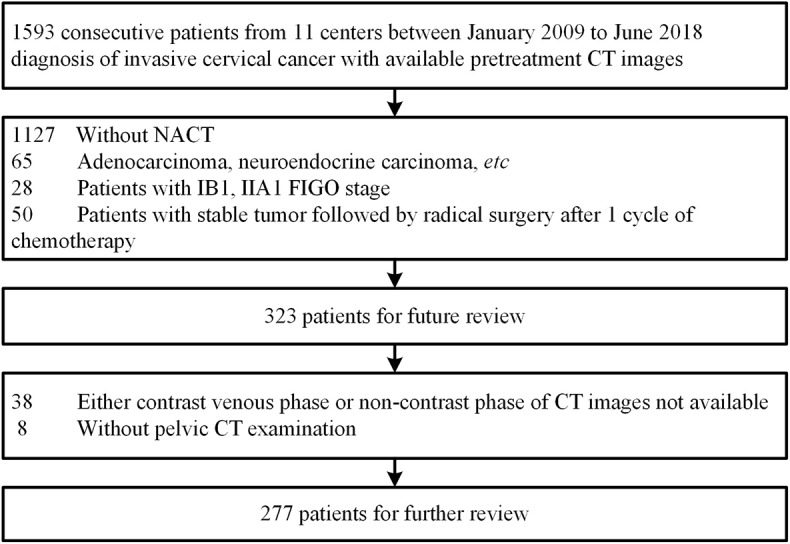
Patient population and exclusions.

This retrospective study was approved by institutional review board of Nanfang Hospital, Southern Medical University with informed consent obtained from all participants. The study was conducted according to the Declaration of Helsinki and was based on data collected in 10 centers from January 2009 to June 2018. In total, 277 consecutive patients with locally advanced cervical cancer underwent neoadjuvant chemotherapy with pre-treatment CT data were included (Figure 1). Eligibility criteria include: (1) Histologically diagnosis of squamous cell carcinoma of the uterine cervix. (2) Patients with clinical stage IB2–III cervical cancer (FIGO 2009) (24). (3) Initial treatment with NACT followed by radical surgery or radiotherapy without any previous therapy. (4) Pre-treatment pelvic CT images. Exclusion criteria are as follows: (1) Underwent preoperative neo-adjuvant concurrent radiotherapy together with chemotherapy. (2) Patients had other malignancy besides cervical cancer. (3) Patients lacking all necessary CT sequences, either venous phase or non-contrast CT images. (4) Patients received only one cycle of preoperative chemotherapy but had insufficient tumor shrinkage that recognized as “stable disease” by RECIST1.1 Criteria. The enrolled 277 patients were randomly divided into a primary cohort of 221 patients (mean age, 48.75 ± 7.94 years; range, 27–69 years) and an independent validation cohort of 56 patients (mean age, 46.36 ± 9.29 years; range, 27–67 years). Both cohorts have comparable ratios of non-responders versus responders. Baseline clinic-pathologic data including age, FIGO stage, HPV infection status, tumor size, and level of SCC antigen before/after chemotherapy were derived from medical records.

### Neoadjuvant Chemotherapy

All the patients received preoperative neoadjuvant chemotherapy intra-arterially or intravenously. Pre-treatment CT examinations were performed within 1 week before the first cycle of NACT. Tumor volume was carefully assessed by gynecological examination together with either transvaginal ultrasound, pelvic MRI, or CT scan at the start of each chemotherapy cycle and 3 weeks after treatment. Final tumor size was confirmed in postoperative tumor pathology. Compared to pre-treatment evaluation, patients with sufficient tumor shrinkage to qualify for operation underwent radical hysterectomy after one cycle of chemotherapy. Patients with insufficient tumor shrinkage for operation had additional 1–2 cycles of chemotherapy at 21-days intervals. Surgery was performed within 21–28 days after the completion of the last cycle. Radiotherapy was performed for patients who had progressive diseases or adverse side effects due to the toxicity of anti-tumor agents during or after NACT.

At the present study, multicentric patients had distinct courses of NACT. Thirty-six patients received only one cycle of intra-arterial chemotherapy, which cisplatin (60–75 mg/m^2^)/carboplatin (300 mg/^2^)+bleomycin (45 mg) was the most adopted program(61.1%). Two hundred and forty-one patients underwent platinum-based combination chemotherapy intravenously, consisting of 1 (103 cases), 2 (124 cases), or 3 (14 cases) courses of treatment. Two hundred and eleven of Two hundred and forty-one (87.5%) patients were treated with paclitaxel (135–175 mg/m^2^) or docetaxel (60–70 mg/m^2^) plus any kinds of platinum regimens. Details are presented in Table A6.

### Assessment of Response

The short-term response was evaluated by change in tumor size according to Response Evaluation Criteria In Solid Tumors (RECIST v. 1.1) as follows: complete response (CR) was defined as eradication of cervical lesion; partial response (PR) (*a* ≥30% decrease in the longest tumor diameter); progressive disease (PD) (*a* ≥20% increase in the longest diameter (LD) of tumor) and stable disease (SD) as the decrease or increase of LD of the cervical lesion was less than PR or PD (25). Many studies proposed two cycles of NACT were adequate for LACC patients to obtain optimal efficacy with relatively low adverse reactions (9, 26). Based on various treatment courses in the study, we set up new response evaluation criteria especially for upcoming radiomic analysis as following: For patients with CR or PR by RECIST (v.1.1) within two cycles of treatment are recognized as “responder” (chemo-sensitive). Whereas, those still with PD or SD after two cycles of chemotherapy were deemed as “non-responder” (chemo-insensitive) regardless of final treatment cycles. Thus, there were 201 responders and 76 non-responders among the qualified 277 consecutive patients (Table A1).

### CT Feature Extraction and Selection

Pre-treatment non-contrast and venous contrasted enhanced CT images at 1.5–3 mm thickness for each case were retrieved from the image-archiving workstation. The whole tumor volume of each patient was manually segmented as region of interest (ROI) by a radiologist via the ITK-SNAP software (www.itksnap.org). As contour of large tumor mass were better visible on contrast-enhanced images. ROI were first drawn on each transverse slice of pelvic venous phase images then applied to non-contrast images. The reproducibility of extracted features from ROIs were ensured through inter- and intra-observer reproducibility evaluation (detailed in the Appendix 1).

In order to get a standard normal distribution of image intensities, each slice of CT images was normalized with z-score. Features were extracted from ROIs of non-contrast and venous enhanced CT using MATLAB R2016b software, respectively. Four groups of features were extracted for each modality: 17 first order statistical features, 8 shape features, 54 texture features and 568 wavelet features (Appendix 2; Table A3). The extracted features are reproducible and match the benchmarks of IBSI (27, 28). Ultimately, a total of 1,294 features were extracted from each patient. Each feature of patients was also normalized with z-score to remove the effect of different CT scanners.

The strategy of feature selection was as follow: Firstly, we selected features with an inter-class correlation coefficients (ICC) greater than 0.85, which usually have good reproducibility. Then, five methods were used to further select predictive features of response, including recursive feature elimination based on a support vector machine (SVM-RFE), least absolute shrinkage and selection operator (LASSO), extremely randomized trees (ET), random forest (RF), and ridge regression. The parameters of each method were determined by grid search with five-fold cross-validation. Ultimately, we selected ones that were repeatedly appeared as the most predictive features among all five methods to be the optimal radiomic features. Feature selection was first performed in the primary cohort and stored the name of the selected features. Then, we selected the features with stored names in validation cohort.

### Building and Validation of Radiomic Signature

For constructing radiomics signature, a RF model was used to predict NACT response. The model was trained on the primary cohort, and parameters of the model were set by grid search with five-fold cross-validation. In the process of training the model, the synthetic minority over-sampling technique (SMOTE) was applied to expand the ratio between responders and non-responders to 1, so that the model was not affected by the sample rate and does not bias to either side during the learning experience. The association of radiomic features with the response of NACT was first assessed in primary cohort, and then validated in validation cohort.

### Construction and Validation of Combined Model

To verify whether prediction performance will be improved when adding clinical information, a multivariable logistic regression model was constructed combining radiomics signature with clinical factors. Clinical candidates include age, FIGO stage and maximum diameter of tumor. Akaikes information criterion (AIC) was adopted to select optimal clinical factors.

After completing model construction, a nomogram was employed to provide a quantitative tool to predict individualized probability of response. For assessing the performance of the model, calibration curves were applied to assess capabilities in primary and validation cohorts, where the curves were used to describe the difference in predictive and actual probability of response. Hosmer-Lemeshow test was adopted to calculate *p*-value to represent the fit degree of predictive and actual curves. Besides, clinical usefulness of the combined model is determined by decision curve analysis. It is created by plotting net benefit at various threshold probability.

**Figure 2:**
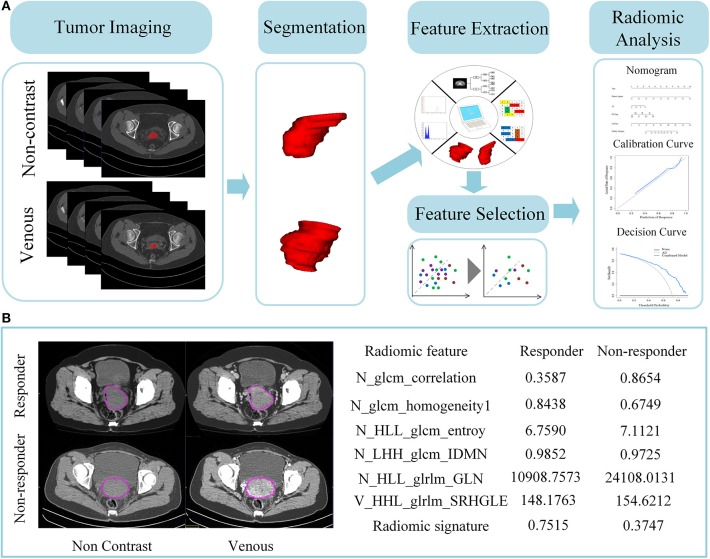
A schema for radiomics pipeline. **(A)** Flowchart of the study. One thousand and twenty-four features were extracted from pre-therapy CT scans. Both extracted radiomic features of non-contrast and venous enhanced images are pooled as part of following feature selection analysis. After chosen by five different feature selection methods, the final top predictive features were then constructed as radiomics signature by Random Forest. Finally, radiomics signature and clinical factors were combined into a nomogram. **(B)** Difference of quantitative radiomic features and predictive probability between chemotherapy responder vs. non-responder. Left images are their corresponding pre-therapeutic CT non-enhanced and venous enhanced images. ROIs are drawn in a purple circle. Right graph indicated the value of radiomic features and predictive probability derived from radiomics signature.

For comparing the performance between the combined model and radiomics signature, Integrated Discrimination Improvement (IDI) was calculated to quantificationally describe the increment of accuracy between the two models. Study flowchart is displayed in Figure 2A.

### Statistical Analysis

The process of statistical analysis was performed on R software (version 3.5.1; https://www.r-project.org). *P* < 0.05 was considered to indicate a statistically significant difference and the *p*-value was bilateral.

## Results

### Clinical Characteristics

Patient characteristics in primary and validation cohorts are given in Table 1. Of the 277 enrolled patients, there were 201 responders (72.56%) and 76 non-responders (27.43%). Median age of the patients was 48 [40–56] years. There are no significant differences between the two cohorts in age and maximum tumor diameter. Primary cohort consisted of 160 responders (72.40%) and 61 non-responders (27.60%) while validation cohort had 41 responders (73.21%) and 15 non-responders (26.79%). CT based LN metastasis positivity was 28.75 and 48.78% in primary and validation cohorts, respectively. Each cohort has a relative equal ratio of different FIGO stages.

**Table 1:** Characteristics of patients in the primary and validation cohorts.

**Characteristics**	**Primary cohort**		***p***	**Validation cohort**		***p***
	**Responder**	**Non-responder**		**Responder**	**Non-responder**	
Age, mean ± SD, years	48.75 ± 7.94	47.87 ± 8.56	0.244	46.80 ± 9.29	43.60 ± 9.72	0.264
**FIGO STAGE**						
IB2	65 (40.63%)	13 (21.31%)	0.010^*^	19 (46.34%)	2 (13.33%)	0.064
IIA2	45 (28.13%)	17 (27.87%)		14 (34.15%)	7 (46.67%)	
IIB-III	50 (31.25%)	31 (50.82%)		8 (19.51%)	6 (40.00%)	
total	160 (72.40%)	61 (27.60%)		41 (73.21%)	15 (26.79%)	
Maximum tumor diameter (cm)	5.13 ± 0.97	5.12 ± 1.35	0.111	5.10 ± 0.81	5.42 ± 0.98	0.209
CT-reported lymphatic status (%)	0.001^*^			0.763
LN-positive	46 (28.75%)	33 (54.10%)		20 (48.78%)	8 (53.33%)	
LN-negative	114 (71.25%)	28 (45.90%)		21 (51.22%)	7 (46.67%)	

*Fisher Exact tests or Chi-Square were applied to compare the differences in categorical variables (CT-reported lymphatic status, pre-treatment FIGO stage). Two-sample t-test was used to compare the differences in age, pre-treatment maximum diameter of tumor. Abbreviations: LN, lymph nodes detected by CT*.

* *P < 0.05*.

### Feature Selection

**Figure 3:**
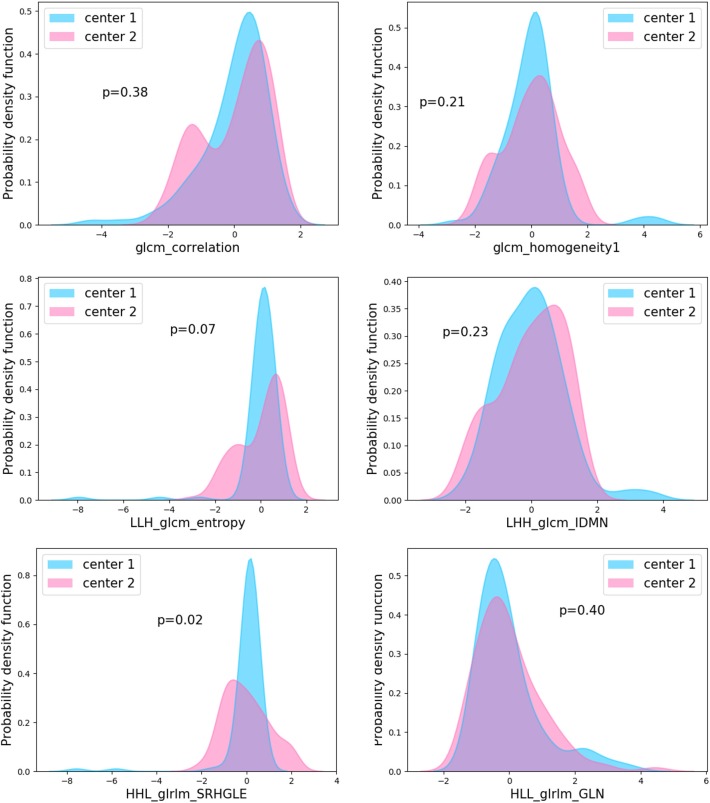
Probability density function of features between centers. Comparison of the six derived features between two hospitals with largest numbers of patients revealed no significant difference exist in the five features from non-contrast CT images (glcm_correlation, LLH_glcm_entropy, HLL_glrlm_GLN, LHH_glcm_IDMN, glcm_homogeneity1) (*P* > 0.05). “HHL_glrlm_SRHGLE” features from venous-enhanced images showed slight difference. P values are for Mann-Whitney *U*-test.

A total of 1294 features were extracted from non-contrast and venous enhanced CT images. Eight hundred and sixty-three features of ICC > 0.85 was reserved for further feature selection. The details of selected features by five methods were presented in Table A4. Finally, six features that repeatedly appeared in five series of the subset of features were selected (Table A5). The comparison of these features between different centers was shown in Figure 3. Because the number of patients is scattered among 10 hospitals, we only selected two hospitals with the largest number of patients for comparison. Results revealed that there are no significant differences in the distribution of five characteristics extracted from non-contrast CT images between hospitals (*P* > 0.05) while the distribution of “HHL_glrlm_SRHGLE” feature derived from venous-enhanced images showed slight difference (*P* = 0.02).

### Construction and Validation of Radiomic Signature

An RF model with Gini criterion was constructed using the top features. The performance of model was first assessed in primary cohort, and then validated in the validation cohort. As shown in Table 2, radiomics signature yielded good performance with an AUC of 0.773 (95% CI, 0.701–0.845) and an accuracy of 78.28% (95% CI, 73.30–83.26%) in primary cohort while achieved an AUC of 0.816 (95% CI, 0.690–0.942) and an accuracy of 80.36% (95% CI, 69.64–89.29%) in validation cohort. There were difference in corresponding quantitative values of radiomic features between non-responders and responders extracted from CT images (Figure 2B).

**Table 2:** Performance of radiomics signature and the combined model.

		**ACC (95% CI)**	**AUC (95% CI)**	**IDI (95% CI)**	***p*-value**
Primary cohort	Radiomics signature	78.28% (73.30–83.26%)	0.773 (0.701–0.845)	0.102 (0.055–0.150)	2.000e−5
	Combined model	80.54% (76.02–85.07%)	0.803 (0.734–0.872)		
Validation cohort	Radiomics signature	80.36% (69.64–89.29%)	0.816 (0.690–0.942)	0.168 (0.032–0.304)	1.516e−2
	Combined model	82.14% (73.21–89.29%)	0.821 (0.697–0.946)		

*Radiomics signature composed of 6 selected features and constructed by Random Forest method. The combined model incorporated radiomics signature with clinical factors (FIGO stage and age). ACC represents the accuracy of prediction, where ACC describes the percentage of the number of accurately predictive patients and total patients. AUC is the acronym of the area under the curve. 95%CI means a 95% confidence interval. IDI is acronym of Integrated Discrimination Improvement, which is for depicting the difference of two models*.

### Construction and Validation of Combined Model

**Figure 4:**
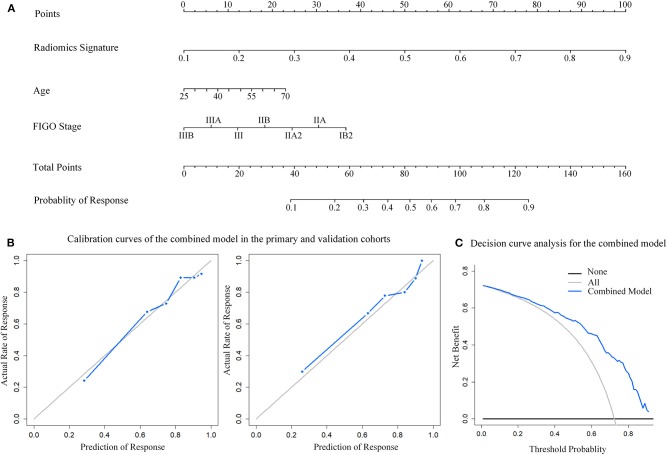
Nomogram developed with the combined model and calibration curves, decision curve analysis for the combined model. **(A)** The developed nomogram. **(B)** Calibration curve of the combined model in the primary and validation cohorts. The middle gray line represents a perfect prediction. The blue line represents the performance of the combined model. Better prediction is demonstrated by a closer fit of the blue line to the grayline. **(C)** Decision curve analysis for the combined model. The y-axis depicts the net benefit. The blue line represents the combined model. The grayline represents the assumption that all patients have response of NACT. The black line is the opposite.

The combined model was constructed incorporating radiomics signature, age and FIGO stage while presented as a nomogram (Figure 4A). The calibration curves of the combined model in both primary and validation cohorts showed a good fit between predictive probability of response and actual response rate (Figure 4B). Non-significant statistics of the accompanied Hosmer-Lemeshow test (primary cohort: *p* = 0.396; validation cohort: *p* = 0.604) implied the model was adequately calibrated without departure from the ideal model. Decision curve demonstrated that the application of the model in clinical decision-making could achieve greater benefits (Figure 4C).

### Comparison of Radiomics Signature and Combined Model

**Figure 5:**
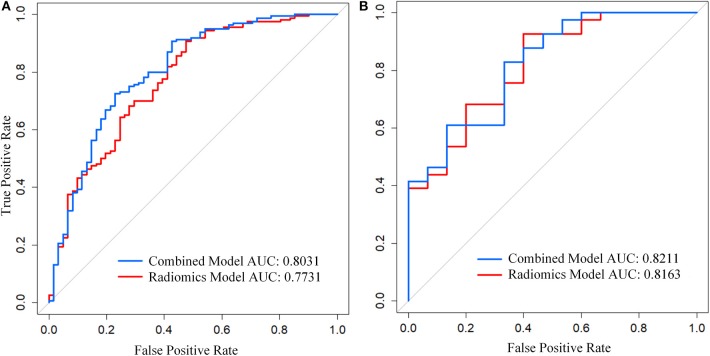
Receiver Operating Curve (ROC) of the models in each cohort. **(A)** ROC in primary cohort. **(B)** ROC in the validation cohort. The middle gray curve represents the dividing line with AUC of 0.5.

The combined model achieved adequate discrimination performance with AUC of 0.803 (95% CI, 0.734–0.872) and AUC of 0.821 (95% CI, 0.697–0.946) in the primary and validation cohorts respectively. In terms of predictive performance, the combined model yielded significant improvement compared to radiomics signature alone (Figure 5; Table 2). Besides, the classification accuracy of the combined model estimating response probability was also superior to sole radiomics signature in both the primary [80.54% (95% CI, 76.02–85.07%)] and validation cohorts [82.14% (95% CI, 73.21–89.29%)], respectively.

## Discussion

In the present study, we successfully established and validated a radiomic signature by pooling together non-enhanced and venous enhanced CT imaging features for predicting response to NACT in LACC using multicenter data set. This six-features radiomics signature can discriminate responders in both the primary (AUC: 0.773) and validation cohort (AUC: 0.816). Moreover, the combined model incorporating radiomics signature and two clinical factors (age, FIGO stage) achieved better performance than radiomic signature alone.

Genomic heterogeneity affect treatment response in LACC. Different responders of neoadjuvant chemotherapy in LACC exhibit diverse genomic profile (29). Twenty-seven-predictive gene signature has been identified to be qualified for distinguishing responders from non-responders in LACC patients treated with concurrent chemoradiotherapy (30). Previous research argued that radiomics could quantify intra-tumor heterogeneity, reflecting underlying tumor gene-expression patterns through the spatial arrangement of imaging voxels (31). Indeed, in the presented study, the established radiomics signature was comprised of six key features describing intratumor voxel patterns (textures) that non-invasively captured intratumor heterogeneity of different responders. “Gray-level co-occurrence matrix” (GLCM) -correlation is a measure of linear dependency of grayscale while GLCM-homogeneity measures local gray level uniformity of the image. As shown in the Figure 2B, local gray level of responder's images is relatively uniform with a larger value of GLCM-homogeneity while signal-intensity variations seem more apparent in non-responder images. “glrlm_GLN” feature is informative for gray level non-uniformity, which shows significant higher value in chemotherapeutic non-responder (32). Note that radiomic features (LHH_glcm_IDMN, LLH_glcm_entropy, HHL_glrlm_SRHGLE) were generated from decomposing original images through using a coiflet wavelet transformation, which at multiple scales could further reflect tumor spatial heterogeneity. This quantitative radiomic features may reflect tumor hetergenous histopathological patterns that are associated with different treatment response such as intratumoral necrosis, vascularization, cellular density (33).

In addition, in order to evaluate the stability and consistency of the established radiomic model, we combined patients from different hospitals to construct two extra radiomic signature as independent validation (Table A2). Only <4% difference was observed between two groups in both training and validation sets (no significant power). Meantime both groups showed good predictive performance, consistent to the model established in the article (Figure A1). This result revealed that our model performed stable between different centers, and it was not affected by patient composition of the training set. Moreover, the reproducibility of derived radiomic features was also robust on a multi-center basis, backed by evidence that after comparison between two hospitals with largest numbers of patients, five of the six features remained stable. Noticeably, the features extracted from non-enhanced CT images were still reliable despite scan-related parameters variation.

Evaluated by AIC methods, adding FIGO stage to radiomics signature enhanced predictive power whereas primary tumor maximal diameter failed. Strikingly, age, a characteristic that rarely proposed to influence treatment response, showing value in model improvement. Consistent with Zhou et al. argument that age might affect NACT efficacy to some extent in cervical cancer (34). The combined model uses a nomogram as a noninvasive and easy-to-use tool for individualized prediction of response to NACT. It simply requires conventional pretreatment CT scans and basic clinical information that readily accessible before the initiation of definitive therapy. Moreover, the features extracted from images in this study were strictly implemented according to the IBSI reference standard to improve the reproducibility.

With its convenience and high predictive accuracy of NACT response, the model could have several clinical implications. The model could facilitate the optimization of individualized LACC initial treatment planning. In fact, according to the identification of chemo-sensitivity, physicians can select chemotherapy-sensitive patients to undergo NACT followed by surgery, whereas, chemotherapy-insensitive patients are subject to radiotherapy instead. This not only benefits young patients in preserving ovarian function and sexual capacity but also improves survival outcome. Recently, NACT has been explored as an approach to fasten the initiation of treatment before access to radiation in some countries with insufficient radiation equipment (35). Radiomic model may assist in identifying qualified patients to undergo NACT before radiation. Moreover, this pre-treatment radiomic model might be of value in predicting response to chemotherapeutic response in metastatic cervical cancer, helping to determine whether alternative treatment approaches might be more appropriate. Further clinical implementation of this convenient model could potentially accelerate the progress of personalized medicine in hope of improving the prognosis of LACC patients.

The major shortcoming in our model is the lack of important clinicopathologic predictive biomarkers such as Ki-67, P53, P protein, especially pretreatment serum SCC antigen. It is largely due to heterogeneous clinical data that was retrospectively collected from different centers. Further researches incorporating pretreatment biomarkers in radiomic model development is needed. Another limitation is that the model can only be effective in squamous cell carcinoma. As various histological type of cervical cancer contributes to distinct chemosensitivity, training cohort only consisted of cases of squamous cell carcinoma for assuring data homogeneity for radiomic analysis. Other histologic subtypes of cervical cancer such as adenocarcinoma, neuroendocrine are unsuitable for model implementation. External validation from a larger dataset of multicentric LACC patients undergoing NACT ought to be applied in justifying the reproducibility and robustness of our proposed radiomic model.

## Conclusion

Pretreatment CT-based radiomic combined model has the best predictive performance for NACT response with radiomic signature remain relatively stable across centers. It may serve as effective and convenient imaging-based predictors that help in patient risk stratification and improved candidate selection for neoadjuvant chemotherapy, facilitating the individualized treatment strategies tailored for LACC patients.

## Data Availability Statement

All datasets for this study are included in the article/Supplementary Material.

## Ethics Statement

This retrospective study was approved by institutional review board of Nanfang Hospital, Southern Medical University with written informed consent obtained from all participants. The study was conducted according to the Declaration of Helsinki.

## Author Contributions

CC and JT: conception and design. XT, WL, HD, LuW, HF, ML, and PLi: collection and assembly of data. CC, PLiu, WL, ZL, CS, XT, HD, and LiW: development of methodology. CS, XT, ZL, WL, HD, LuW, LiW, PLiu, CC, and JT: data analysis and interpretation. All authors: manuscript writing and final approval of manuscript.

### Conflict of Interest

The authors declare that the research was conducted in the absence of any commercial or financial relationships that could be construed as a potential conflict of interest.
