# Supplementary material for: Prediction of Response to Preoperative Neoadjuvant Chemotherapy in Locally Advanced Cervical Cancer Using Multicenter CT-Based Radiomic Analysis
Source: Front Oncol. 2020 Feb 4;10:77. doi: 10.3389/fonc.2020.00077 (PMC7010718; doi:10.3389/fonc.2020.00077)

**Appendix 1: Inter- and Intra-observer reproducibility evaluation**

**Statistical analysis:** Inter- and intra-observer reproducibility of ROI-based extracted features were initially analyzed with the non-contrast and venous CT images of 30 patients (randomly selected). Intra-class correlation coefficients (ICCs) (1) was defined for estimating the inter and intra-observer agreement on the basis of feature extraction. ICC of 0.85-1.00 as almost optimal agreement, 0.61-0.84 as great agreement, 0.41-0.60 as medium agreement, 0.21-0.40 as fair agreement, and 0-0.20 as no agreement. An ICC great than 0.60 was regarded as a satisfactory reproducibility. To ensure the intra-observer reproducibility, each reader repeated the tumour masking and feature extraction twice with an interval of at least 1 month, following the same procedure.

**Result****:** The ICCs of tumour masking and generation of feature on the basis of radiologist’s twice measurements were approach to a satisfying level, ranging from 0.75 to 0.98. The ICCs of comparison between the two radiologists were good (5 years and 10 years of experience), ranging from 0.70 to 0.92.

**Reference**

1. McAlinden C, Khadka J, Pesudovs K (2011) Statistical methods for conducting agreement (comparison of clinical tests) and precision (repeatability or reproducibility) studies in optometry and ophthalmology. Ophthalmic Physiol Opt 31:330–33

**Appendix 2: Radiomic feature defination**

Specific feature extracted are shown in **Appendix Table A1**. Among them, calculation formulas of features were easily founded in previous studies (2,3), thus, we only provide their name. A total of 647 quantitative radiomic features including 8 shape features, 17 first order statistical features, 54 textural features and 568 wavelet features, were extracted respectively from non-contrast CT, and venous CT. We applied undecimated 3-dimensional wavelet transformation (coiflet) to decompose the original image into 8 parts. L and H indicate low- and high-pass functions, respectively. The wavelet decompositions of the original image$X$were labeled as$X_{LLL},X_{LLH},X_{LHL},X_{LHH},X_{HLL},X_{HLH},X_{HHL},X_{HHH}$. For example,$X_{LLH}$represents the image filtered with low-pass function in the x- and y-directions, and high-pass function in the z-direction, as follows:

$$X_{LLH}(i,j,k)=\sum_{p=1}^{Nl} \sum_{q=1}^{Nl} \sum_{r=1}^{Nh} L(p)L(q)H(r)X(i+p,j+q,k+r)$$

Where$Nl$ is the length of filter L, Nh is the length of filter H.

**Reference**

2. Aerts HJWL, Velazquez ER, Leijenaar RTH, et al (2014) Decoding tumour phenotype by noninvasive imaging using a quantitative radiomics approach. Nat Commun. doi: 10.1038/ncomms5006

3. Gillies RJ, Kinahan PE, Hricak H (2016) Radiomics: Images Are More than Pictures, They Are Data. Radiology 278:563–577

**Tables**

**Appendix Table A1** Patient No. of hospitals

| **Hospital** | **Responder** | **Non-responder** | **Patients No.** |
| --- | --- | --- | --- |
| A | 54 | 46 | 100 |
| B | 98 | 12 | 110 |
| C | 4 | 2 | 6 |
| D | 8 | 0 | 8 |
| E | 17 | 2 | 19 |
| F | 8 | 2 | 10 |
| G | 0 | 1 | 1 |
| H | 5 | 0 | 5 |
| I | 7 | 1 | 8 |
| J | 8 | 2 | 10 |

Abbreviations: A, Nanfang hospital; B, Jiangmen Central Hospital; C, Army Daping Hospital; D, Chongqing Army Xinqiao Hospital; E, Affiliated Tumor Hospital of Guangzhou Medical University; F, Henan Provincial People's Hospital; G, Nanshan People's Hospital; H, Ningbo Maternal and Child Health Hospital; I, Yantai Sulphur Top Hospital; J, First Affiliated Hospital of Zhengzhou University.

**Appendix Table A2.** Random combination of hospitals

| **Combination** | **Training set** | | |  | **Validation set** | | |
| --- | --- | --- | --- | --- | --- | --- | --- |
|  | **Hospital** | **Patient No.** | **Positive ratio** |  | **Hospital** | **Patient No.** | **Positive ratio** |
| **Group 1** | B | 110 | 0.89 |  | C, D, E, F, G, H, I, J | 67 | 0.85 |
| **Group 2** | A (-30), E, H, I | 91 | 0.73 |  | B (5), C, D, F, G, J | 51 | 0.73 |

Abbreviations: A, Nanfang hospital; B, Jiangmen Central Hospital; C, Army Daping Hospital; D, Chongqing Army Xinqiao Hospital; E, Affiliated Tumor Hospital of Guangzhou Medical University; F, Henan Provincial People's Hospital; G, Nanshan People's Hospital; H, Ningbo Maternal and Child Health Hospital; I, Yantai Sulphur Top Hospital; J, First Affiliated Hospital of Zhengzhou University.

**Appendix Table A3** Features used in this experiment

| **Category** | | **Feature name** | | | | | | | | |
| --- | --- | --- | --- | --- | --- | --- | --- | --- | --- | --- |
| **Shape and size** | | Compactness1 | Compactness2 | Maximum 3D diameter | Spherical disproportion | Spherical disproportion | Sphericity | Surface area | Surface to volume ratio | Volume |
| **First order statistic** | | Energy | Entropy | Kurtosis | Maximum | Mean | Mean absolute deviation | Median | Minimum | Range |
|  |  | Root mean square | Skewness | Standard deviation | Uniformity | Variance | Sum | Entropy after normalization | Uniformity after normalization |  |
| **Texture** |  |  |  |  |  |  |  |  |  |  |
|  | **GLCM** | Autocorrelation | Entropy | Cluster shade | Homogeneity1 | Homogeneity2 | Correlation | Contrast | Dissimilarity | Energy |
|  |  | Cluster prominence | Cluster tendency | Difference entropy | Information measure of correlation1  (IMC1) | Information measure of correlation2  (IMC2) | Inverse difference moment normalized  (IDMN) | Inverse difference normalized | Inverse variance | Maximum probability |
|  |  | Sum average | Sum entropy | Sum variance | Covariance |  |  |  |  |  |
|  | **GLRLM** | Short run emphasis (SRE) | Long run emphasis (LRE) | Gray level non-uniformity (GLN) | Run length non-uniformity (RLN) | Run percentage (RP) | Low gray level run emphasis (LGLRE) | High gray level run emphasis (HGLRE) | Short run low gray level emphasis (SRLGLE) | Short run high gray level emphasis (SRHGLE) |
|  |  | Long run low gray level emphasis (LRLGLE) | Long run high gray level emphasis (LRHGLE) | Mean | Entropy | Energy |  |  |  |  |
|  | **GLSZM** | Large zone low gray-level emphasis (LZLGE) | Large zone high gray-level emphasis (LZHGE) | Gray-level long-uniformity (GLN) | Zone-size non-uniformity (ZSN) | Small zone high gray-level emphasis (SZHGE) | Low gray-level zone emphasis (LGLZE) | High gray-level zone emphasis (HGLRE) | Small zone low gray-level emphasis (SZLGE) | Zone percentage (ZP) |
|  |  | Small zone emphasis (SZE) | Large zone emphasis (LZE) | Gray-level variance (GLV) | Zone-size variance (ZSV) |  |  |  |  |  |
|  | **NGTDM** | Coarseness | Contrast | Busyness | Complexity | Strength |  |  |  |  |

**Appendix Table A4** Details of selected features by five methods

| **SVM-RFE** | **Lasso** | **ET** | **RF** | **Ridge** |
| --- | --- | --- | --- | --- |
| N_LLH_glcm_entropy | N_glcm_correlation | N_glcm_correlation | V_HHL_glrlm_SRHGLE | N_glcm_homogeneity1 |
| N_LHH_fos_energy | N_glrlm_GLN | N_glszm_ZSNU | N_glcm_correlation | V_HHL_glcm_maximum_probability |
| N_LHH_fos_mass | N_LLL_ngtdm_busyness | N_LHL_glszm_LGLZE | N_HHL_glrlm_HGLRE | N_LHH_glszm_LGLZE |
| N_LHH_glcm_IDMN | N_LLL_glszm_GLNU | N_LHH_glcm_energy | N_LHH_glcm_IDMN | V_LLH_glcm_energy |
| N_LHH_glrlm_energy | N_LLH_glcm_entropy | N_HLL_glrlm_GLN | N_LLL_glcm_correlation | N_HLH_glrlm_GLN |
| N_HHL_fos_krutosis | N_LHL_glszm_LZSE | N_HHL_glcm_dissimilarity | N_HHH_glcm_IDN | V_LHH_glcm_inverse_variance |
| N_HHL_fos_variance | N_HLL_glrlm_GLN | V_glcm_difference_entropy | V_LHH_fos_energy | N_HHL_glrlm_GLN |
| N_HHH_glrlm_RLN | N_HLH_glrlm_entropy | V_HLL_fos_standard_deviation | N_HLL_glrlm_GLN | N_HHH_glcm_homogeneity1 |
| V_LLH_fos_entropy_p |  |  |  |  |
| V_HLL_glcm_difference_entropy |  |  |  |  |

Abbreviations: SVM-RFE, recursive feature elimination based on a support vector machine; Lasso, least absolute shrinkage and selection operator; ET, extremely randomized trees; RF, random forest; N, non-contrast CT images; V, venous CT images; H and L is the direction of wavelet decompositions.

**Appendix Table A5 Details of final selected features**

| Sequence | Feature name | Phase | Category |
| --- | --- | --- | --- |
| Non-contrast |  |  |  |
|  | glcm_correlation | initial | texture |
|  | LLH_glcm_entropy | wavelet | texture |
|  | HLL_glrlm_GLN | wavelet | texture |
|  | LHH_glcm_IDMN | wavelet | texture |
|  | glcm_homogeneity1 | initial | texture |
| Venous |  |  |  |
|  | HHL_glrlm_SRHGLE | wavelet | texture |

Abbreviations: H and L is the direction of wavelet decompositions

**Appendix Table A6. Main regimens of NACT**

|  | **Primary cohort** | | **Validation cohort** | |
| --- | --- | --- | --- | --- |
| **Intra-arterial chemotherapy** | number | cycles | number | cycles |
| Cisplatin/carboplatin plus bleomycin | 17 | 1 | 5 | 1 |
| Paclitaxel/docetaxel plus cisplatin/carboplatin/Lobaplatin | 9 | 1 | 3 | 1 |
| Platinum monotherapy | 1 | 1 | 1 | 1 |
| Total | 27 |  | 9 |  |
|  |  |  |  |  |
| **Intravenous chemotherapy** |  |  |  |  |
| Flfluorouracil plus cisplatin or irinotecan plus carboplatin | 8 | 1~3 | 0 |  |
| Paclitaxel plus cisplatin/carboplatin/Oxaliplatin/ nedaplatin | 168 | 1~3 | 43 | 1~3 |
| Docetaxel plus nedaplatin/Oxaliplatin/cisplatin | 18 | 1~3 | 4 | 1~2 |
| Total | 194 |  | 47 |  |

**Appendix Figure A1** ROC curves of groups in training (left) and validation (right) sets.


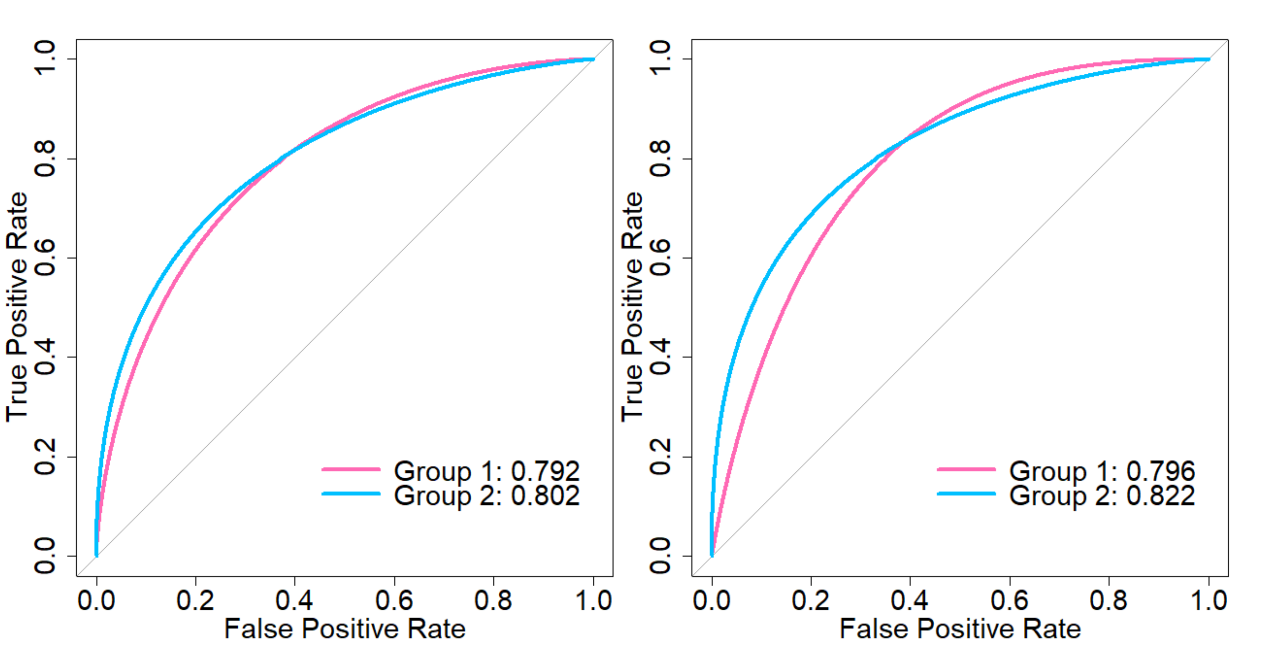

Supplement: Supplementary file 1 [file Table_1.docx]
